# Prognostic role of indoleamine 2,3-dioxygenase 1 expression in solid tumors: A systematic review and meta-analysis

**DOI:** 10.3389/fonc.2022.954495

**Published:** 2022-09-23

**Authors:** Haiyan Zhang, Jing Li, Qi Zhou

**Affiliations:** ^1^ Pharmaceutical Department, Hubei Cancer Hospital, Tongji Medical College, Huazhong University of Science and Technology, Wuhan, China; ^2^ School of Pharmaceutical Sciences, Guangzhou University of Chinese Medicine, Guangzhou, China

**Keywords:** IDO1, tumor, prognosis, survival, meta-analysis

## Abstract

**Background:**

As an emerging immune checkpoint molecule, indoleamine 2,3-dioxygenase 1 (IDO1) is an immunosuppressive rate-limiting enzyme in metabolism of tryptophan to kynurenine. The expression of IDO1 affected the prognosis of patients in cancers by regulating the kynurenine pathway, inhibiting the proliferation of T cells. However, the association between IDO1 and solid tumor prognosis was controversial. To further investigate the role of IDO1 expression in solid tumors, we conducted the systematic review and meta-analysis.

**Methods:**

We searched the Web of Science, PubMed, Embase, and Cochrane Library databases and China National Knowledge Infrastructure (CNKI) to identify studies evaluating the prognostic value of IDO1 in solid tumors. Overall survival (OS), progression-free survival (PFS), and disease-free survival (DFS) were extracted as the outcome. Pooled hazard ratios (HRs) with 95% confidence intervals (CIs) were calculated by using the fixed-effect/random-effect model, while heterogeneity, publication bias, and sensitivity between studies were also analyzed.

**Results:**

Eighteen studies with 2,168 patients were included in this systematic review and meta-analysis. The results indicated that the high expression of IDO1 was associated with a shorter OS (n = 1926, HR = 1.60, 95% CI: 1.22–2.11, P = 0.001) and DFS (n = 327, HR = 2.65, 95% CI: 1.52–4.63, P = 0.001), while it was uncorrelated with PFS (n = 428, HR = 1.76, 95% CI: 0.99–3.14, P = 0.240). There was significant heterogeneity between studies on OS (I^2^ = 77.8%, P < 0.001). Subgroup analysis showed that age, gender, tumor type, follow-up period, and study quality were possible reasons for high heterogeneity. The result of the trim-and-fill method indicated that publication bias for OS had no impact on our results. Egger’s test suggested no publication bias for PFS (P = 0.553) and DFS (P = 0.273). Furthermore, sensitivity analysis indicated the result was stable.

**Conclusion:**

High expression of IDO1 was associated with poor clinical outcomes, indicating that it could be a potential prognostic marker in various cancer types.

## Introduction

In the last decade, cancer immunotherapy has made significant progress with the application of immune checkpoint inhibitors (ICs). Cytotoxic T-lymphocyte-associated antigen 4 (CTLA-4), programmed death 1 (PD-1), and programmed cell death 1 ligand 1 (PD-L1) inhibitors had shown good safety and efficacy in various tumor types, including genitourinary oncology, gastrointestinal oncology, and hematologic tumor (1). It is reported that high mutational burden and PD-L1 expression of urothelial carcinoma made it more sensitive to immunotherapy (2, 3), and several ICs had been approved to treat it (4). Similarly, ICIs had also shown good clinical efficacy in prostatic cancer (5), penile cancer (6), testicular cancer (7), colorectal cancer (8), and esophagus cancer (9). Additionally, nivolumab, pembrolizumab, camrelizumab, and tislelizumab had been approved to treat Hodgkin lymphoma (HL) (10).

With the success of PD-1, PD-L1, and CTLA-4 inhibitors, more and more emerging new immune checkpoints attract extensive attention, including lymphocyte activation gene 3, T-cell immunoglobulin- and mucin-domain-containing molecule 3, T-cell immunoglobulin and ITIM domain, and indoleamine 2,3-dioxygenase 1(IDO1) (11). IDO1, an intracellular enzyme, plays a vital role in converting tryptophan into downstream kynurenines, which could suppress T-cell proliferation *in vitro*, induce the T-cell apoptosis, and affect the natural killer (NK) cell function by depleting the tryptophan (12–14). In normal physiological conditions, IDO1 is mainly expressed in mucosal tissues, placenta, eye, pancreas, and some immune cell subsets (15). In tumors, high expression of IDO1 could cause overactivation of the kynurenine pathway, which could suppress the effector cells and promote the activation of the immunosuppressive cells, consequently forming an immunosuppressive environment in tumors (13, 14, 16). It was reported that IDO1 was an immune checkpoint which could be potentially exploited to improve treatment outcomes in various cancers (17, 18). Additionally, a large number of IDO1 inhibitors had been developed as anticancer drug in clinical trials, including NLG-8189, INCB024360, GDC-0919, PF-06840003, and BMS986205 (12, 18).

Numerous studies have reported that IDO1 is expressed and correlated with prognosis in various cancers, such as urothelial bladder cancer (17), non-small–cell lung cancer (NSCLC ) (19), esophageal squamous cell carcinoma (20), breast cancer (21), pancreatic cancer (22), endometrial cancer (23), and neuroendocrine skin cancer (24). However, the role of IDO1 in clinical cancer studies is still conflicting. Chen et al. reported that the overexpression of IDO1 was associated with poor survival of patients with diffuse large B-cell lymphoma (25). Inversely, the study of Ma et al. showed that high IDO1 expression was significantly associated with better overall survival (OS) for patients in adenosquamous lung carcinoma (26). It was also reported that the high expression of IDO1 was positively correlated with other immune checkpoints (17, 19, 27–29). Vienna’s study showed that high programmed cell death 1 ligand 2/IDO-1 co-expression levels were independent negative prognostic factors for survival in early NSCLC. Wei’s study suggested that progression-free survival (PFS) was associated with IDO/Foxp3 co-expression levels in breast cancer. In addition, many factors could have an effect on the expression of IDO1, such as pathological type, virus infection, and therapeutic straregies (14, 30). For example, triple-negative breast cancer (TNBC) patients were confirmed to have a higher IDO1 expression compared to non-TNBC patients (14). Higher IDO1 expressions were also found in human papillomavirus-positive head and neck squamous cell carcinoma patients (30) and hepatitis B virus-positive liver parenchymal cells (31). In terms of therapeutic straregies, chemotherapy regimens could regulate the immune microenvironment by inducing a period of transient lymphopenia and homeostatic recovery and further affecting the expression of IDO1 (32). Thus, it is necessary to investigate the correlation between IDO1 expression and tumor prognosis. Meanwhile, not only IDO1 expression but also single-nucleotide polymorphisms of IDO1 (rs9657182, rs3739319) were associated with outcome in tumor patients (33, 34).

The previous meta-analysis only involved studies prior to 2019 (35, 36). In recent years, numerous studies have been published related to IDO1 in cancer patients. Additionally, the expression of IDO1 in tumor tissue could be affected by different treatment methods, such as chemotherapy and immunotherapy. The previous meta-analysis ignored the effect of treatment before surgery or biopsy. In the systematic review, we would analyze the association of IDO1 expression in treatment-naive tissue with cancer survival.

## Method

### Literature search

We searched all relevant literature in Embase, PubMed, the Cochrane Library, Web of Science, and China National Knowledge Infrastructure (CNKI) on 3 April 2022, without language restriction. The keywords, including indoleamine 2,3-dioxygenase 1, tumor, prognosis, survival, treatment outcome, and their medical subject headings (MeSH) terms, were used to build a search strategy. This meta-analysis was carried out based on the Preferred Reporting Items for Systematic Reviews and Meta-Analyses (PRISMA) statement.

### Study selection

Our systematic review addressed the following research question: In cancer patients, what is the relationship between IDO1 expression and cancer survival? The established Patients, Intervention, Comparison, Outcomes, and Study design (PICOS) framework was employed to the selection criteria. The PICOS strategy was defined as follows: “P” (patient)—patients with tumor, “I” (intervention)—not applicable, “C” (comparison)—comparison with a high expression group or positive groups according to IDO1 expression, “O” (outcome)—relevant indicators to evaluate the association between expression of IDO1 and prognostic outcomes in tumor tissue, and “S” (study design)—prospective studies, and retrospective study.

According to the PICOS principles, the inclusion criteria were as follows: 1) studies evaluated the association between expression of IDO1 and prognostic outcomes in tumor tissue; 2) the patients were divided into high and low expression groups, or positive and negative groups according to expression level of IDO1; 3) studies reported the prognosis of IDO1 expression using Kaplan–Meier survival analysis, univariate Cox regression analysis, or multivariate Cox regression analysis.

The exclusion criteria were as follows: 1) reviews, case reports, letters, editorials, meeting abstracts; 2) full text was not available; 3) animal experiments or *in vitro* experiments rather than clinical studies; 4) IDO1 expression was not detected in tumor tissues; 5) IDO1 expression was only detected in RNA level by reverse transcription-polymerase chain reaction (RT-PCR); 6) patients who received antitumor therapy before surgery or biopsy or unclear; 7) hazard ratios (HR) and 95% confidence intervals (CI) were not directly provided.

### Data extraction

Three investigators independently extracted the data according to the same criteria, and disagreement between the three investigators would be resolved by consensus. The collected data included publication year, the name of first author, country, tumor type, median of age, gender, number of patients, treatment method, detection method for IDO1 expression, outcome endpoints, median of follow-up period, and the method to estimate HRs and CIs. OS, progression-free survival (PFS), and disease-free survival (DFS) were chosen as outcome endpoints.

### Quality assessment

Three investigators independently assessed the quality of included studies by the Quality In Prognostic Studies (QUIPS) tool (37, 38). Study participation, study attrition, prognostic factor measurement, outcome measurement, study confounding, and statistical analysis were assessed. If more than four of these six criteria had a low risk of bias, the study was considered to be low risk of bias, and if two or more criteria had a high risk of bias, the study was considered to be high risk of bias. The remaining studies were classified as moderate risk of bias.

### Statistical analysis

Stata version 14.0 (Stata Corporation, College Station, TX, USA) was used to carry out the statistical analysis. Pooled HRs and 95% CIs for OS, PFS, and DFS were used to assess the association between IDO1 expression and survival. Heterogeneity was assessed by the I^2^ value derived from the Q test. We considered P < 0.05 or I^2^ > 50% as significant heterogeneity. According to the I^2^ and P values, different effect models were used. When I^2^ > 50% or P < 0.05, a random-effect model was used. Otherwise, we used a fixed-effect model. Egger’s test and trim-and-fill method were used to assess publication bias. Sensitivity analysis was used to assess the stability of results by excluding one study.

## Results

### Study selection

**Figure 1:**
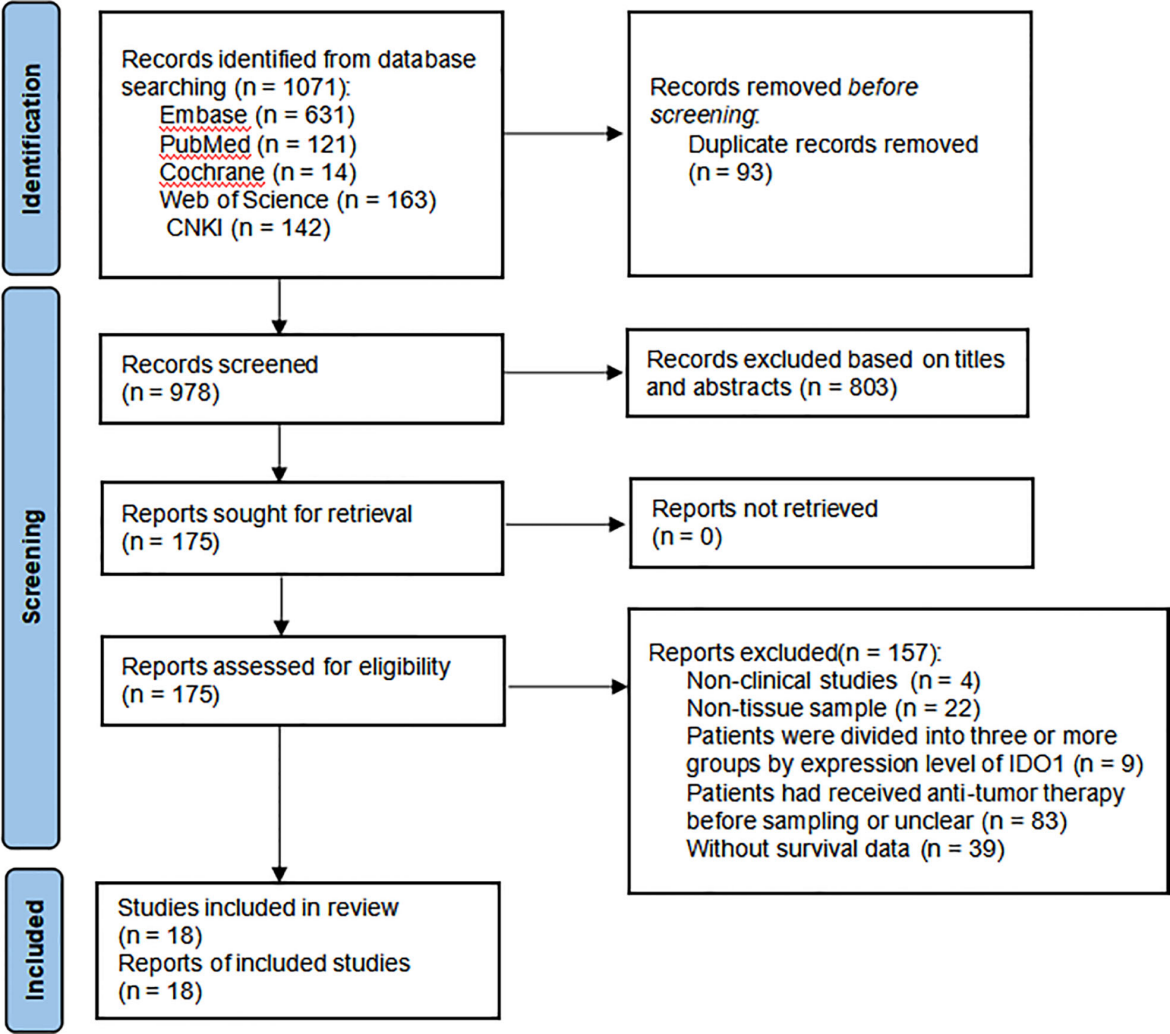
Flow diagram of the study selection.

A total of 1,071 papers were initially identified. After removing duplicate literature and reading the title, abstract, and full text according to the study’s inclusion and exclusion requirements, inappropriate publications were excluded. Finally, 18 studies were identified, including 2,168 patients with malignant tumors (17, 25, 28, 39–53). These studies were published between 2008 and 2020. The flow diagram for the study selection is presented in **Figure 1**.

### Study characteristics and quality assessment

The characteristics of the studies involved in this meta-analysis are shown in **Table 1**. The study subjects were from Asia (China: 10, Korea: 2, Japan: 3, n = 15) and non-Asia (Tunisia: 2, USA: 1, n = 3). Cancer types included breast cancer (n = 4), bladder cancer (n = 1), diffuse large B-cell lymphoma (n = 2), nasopharyngeal carcinoma (n = 2), hepatocellular carcinoma (n = 3), vulvar squamous cell carcinoma (n = 1), esophageal squamous cell cancer (n = 1), HL(n = 1), laryngeal squamous cell carcinoma (n = 1), osteosarcoma (n = 1), and endometrial cancer (n = 1). The sample size of the included studies ranged from 47 to 362. There were 15 studies evaluating the effect of IDO1 expression on OS, six studies on DFS, and three on DFS. The expression of IDO1 was detected by immunohistochemistry (IHC), RT-PCR, Western blot (WB), quantitative immunofluorescence (QIF), or computational pathology analysis (CPA). Almost all the patients received chemoradiotherapy during the follow-up period. HRs were estimated by multivariate regression analysis in most studies (n = 16). According to the QUIPS checklist, seven studies had an overall low risk of bias and 11 studies had an overall moderate risk of bias.

**Table 1:** Characteristics of studies included in the meta-analysis.

Year	First author	Country	Cancer type	Age median (range, years)	Male/female	Sample size	Treatment	Outcome	Detection method	Type of study	Follow-up period (years)	Multivariate	Risk of bias
2020	Lijuan Wei	China	BC	49 (30–79)	0/77	77	NA	PFS	IHC	R	NA	Yes	M
2020	Donghyun Kim	Korea	BCA	NA	NA	69	Radiotherapy	OS, DFS	IHC	R	NA	Yes	M
2020	Xiangli Chen	China	DLBCL	56 (24-81)	42/18	60	Chemoradiotherapy	OS, PFS	IHC	R	2.91	Yes	L
2019	Ya Qin Wang	China	NPC	49.98	55/153	208	Chemoradiotherapy	OS	IHC,CPA	R	5.8	No	M
2019	Yan Wang	China	HCC	NA	129/24	153	NA	OS	IHC	R	≥8	Yes	M
2019	Nadia Boujeleben	Tunisia	VSCC	65.6 (35-91)	NA	61	NA	OS, PFS	IHC	R	≥5	Yes	L
2019	Lu Xiaoting	China	BC	51 (30-75)	0/100	100	NA	PFS	IHC	R	4.7	Yes	M
2018	Lijuan Wei	China	BC	52 (30-79)	NA	65	NA	PFS	IHC	R	5.7	Yes	M
2017	Daniel E. Carvajal-Hausdorf	USA	BC	NA	0/100	362	NA	OS	QIF	R	≥20	Yes	L
2016	Ahlem BenHajAyed	Tunisia	NPC	47 (8-80)	2.08	71	NA	OS, DFS	IHC, RT-PCR	R	2.5	Yes	L
2015	Yunlong Ji	China	ESCC	54 (29-73)	132/64	196	NA	OS	IHC, RT-PCR	R	≥5	No	L
2014	JiYoung Choe	Korea	HL	NA	NA	97	Chemoradiotherapy	OS	IHC	R	7	Yes	M
2013	Jin Ye	China	LSCC	52.4 (37-85)	179/8	187	Chemoradiotherapy	OS, DFS	IHC	R	≥5	Yes	M
2011	Soranobu Ninomiya	Japan	DLBCL	NA	67/52	119	Chemotherapy	OS	IHC	R	≥3	Yes	L
2009	Wang Jin	China	HCC	46.9 (30-70)	79/14	93	NA	OS	IHC	R	1.58	Yes	M
2009	Hiroshi Urakawa	Japan	Osteosarcoma	NA	30/17	47	NA	OS	IHC, WB	R	5.61	Yes	M
2008	Ke Pan	China	HCC	NA	118/20	138	NA	OS	IHC	R	≥5	Yes	M
2008	Kazuhiko Ino	Japan	EA	57.7	NA	65	Chemoradiotherapy	OS, PFS	IHC	R	6	Yes	L

PSCC, penile squamous cell carcinoma; BC, breast cancer; BCA, bladder cancer; DLBCL, diffuse large B-cell lymphoma; NPC, nasopharyngeal carcinoma; HCC, hepatocellular carcinoma; VSCC, vulvar squamous cell carcinoma; ESCC, esophageal squamous cell cancer; HL, Hodgkin lymphoma; LSCC, laryngeal squamous cell carcinomas; EA, endometrial cancer; DSS, disease-specific survival; PFS, progression-free survival; OS, overall survival; DFS, disease-free survival; IH, immunohistochemistry; WB, Western blot; CPA, computational pathology analysis; QIF, quantitative immunofluorescence; RT-PCR, reverse transcription-polymerase chain reaction; R, retrospective study; L, low-risk; M, moderate-risk; NA, not available.

### The relation between IDO1 expression and OS in solid tumor patients

**Figure 2:**
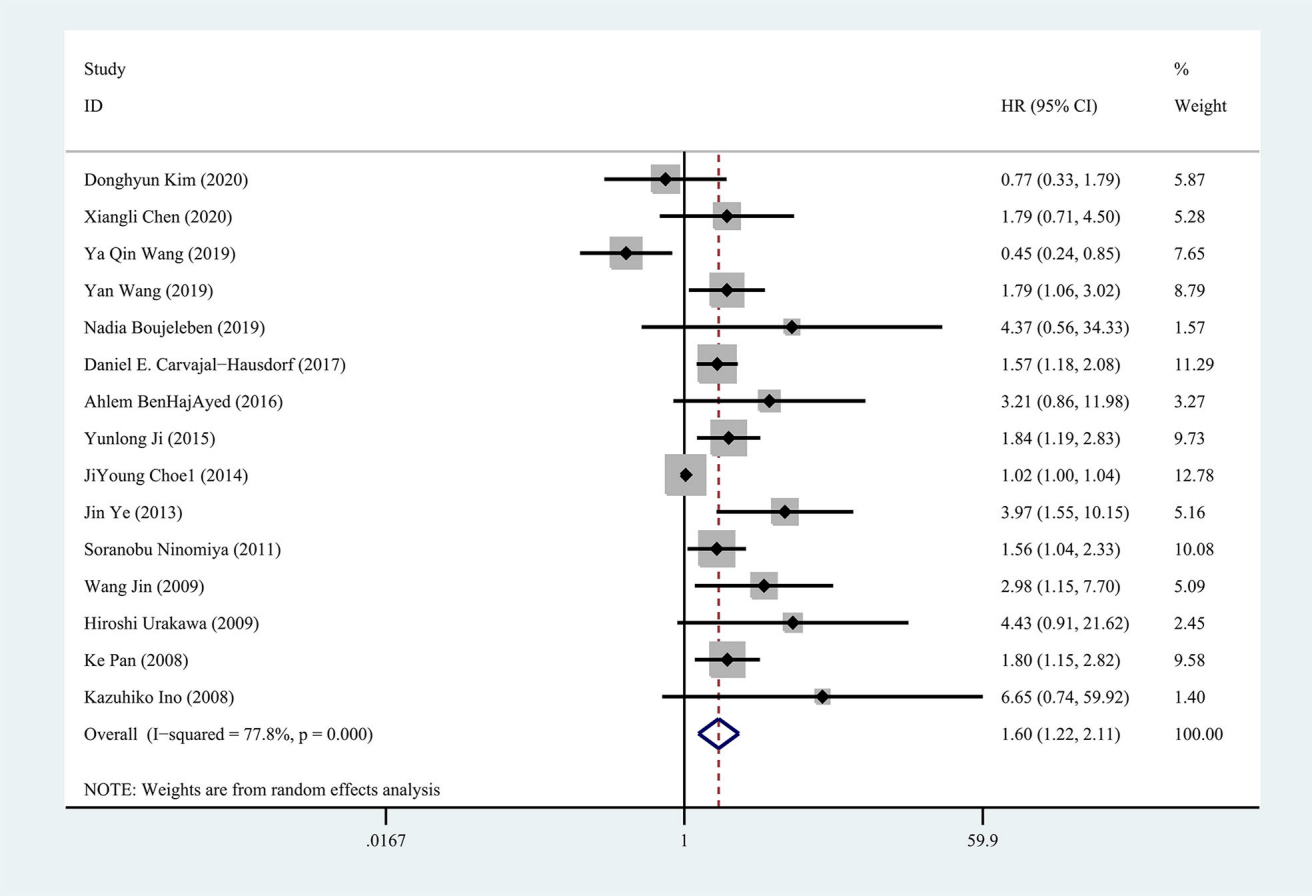
Forest plot of HR with 95% CI for correlation between expression of IDO1 and overall survival.

Fifteen studies were included in the meta-analysis of OS. A random-effect model was used to calculate the pooled HRs and 95% CIs, as the heterogeneity test reported a P value of 0 and an I^2^ value of 77.8%. The results showed that patients with a higher expression of IDO1 had a significant shorter OS (n = 1926, HR = 1.60, 95% CI: 1.22–2.11, P = 0.001) (**Figure 2**). Subgroup analyses of OS were further carried out according to multiple potential factors (age, gender, sample sizes, ethnicity, tumor type, detection method, follow-up periods, method to estimate HR, and study quality) to investigate the heterogeneity (**Table 2**). The result of the subgroup analysis showed that age, gender, follow-up period, tumor type, and the quality of the included studies were possible reasons for the high heterogeneity. The heterogeneity of age <50 (HR = 1.52, 95% CI: 0.36–6.44, P = 0.567), male/female <1 (HR = 1.35, 95% CI: 0.49–3.70, P = 0.559), and follow-up period ≥5 (HR = 1.58, 95% CI: 1.12–2.22, P < 0.05) were as high as I^2^ = 85.9%, I^2^ = 88.7%, and I^2^ = 81.7%, respectively, while no heterogeneity was found for age ≥50 (HR = 2.16, 95% CI: 1.52–3.07, P < 0.001), male/female >1 (HR = 1.81, 95% CI: 1.47–2.23, P < 0.001), and follow-up period <5 (HR = 1.80, 95% CI: 1.29–2.51, P < 0.05). In terms of tumor type, IDO1 expression was not associated with OS in nasopharyngeal carcinoma (n = 2, I^2^ = 85.6%, P = 0.922) but was related to worse OS in diffuse large B-cell lymphoma (n = 2, I^2^ = 0, P < 0.05) and hepatocellular carcinoma (n = 3, I^2^ = 0, P < 0.001). Subgroup analysis of other cancer types was not performed because only one study was involved. Studies with low-risk quality showed a tendency to increase the risk of shorter OS (HR = 1.69, 95% CI: 1.39–2.05, P < 0.001) without heterogeneity. In addition, the results of subgroup analyses by sample size, ethnicity, and detection method showed that the high expression of IDO1 was correlated with worse OS.

**Table 2:** Subgroup analysis of OS included in the meta-analysis.

Subgroup	HR (95% CI)	P	Heterogeneity		Studies
			P	I^2^	
**Age**					
≥50	2.16 (1.52-3.07)	<0.001	0.438	0	5
<50	1.52 (0.36-6.44)	0.567	<0.05	85.9%	3
NA	1.43 (1.07-1.93)	<0.05	0	78.3%	7
**Male/female**					
>1	1.81 (1.47-2.23)	<0.001	0.887	0	7
<1	1.35 (0.49-3.70)	0.559	<0.001	88.7%	3
NA	1.49 (0.77-2.89)	0.242	0.077	52.6%	5
**Sample size**					
>97	1.60 (1.22-2.11)	<0.05	<0.001	77.8%	7
≤97	1.84 (1.08-3.13)	<0.05	0.013	60.5%	8
**Ethnicity**					
Asia	1.55 (1.13-2.12)	<0.05	<0.001	77.7%	12
Non-Asia	1.65 (1.25-2.17)	<0.001	0.376	0	3
**Cancer type**					
DLBCL	1.59 (1.10-2.30)	<0.05	0.786	0	2
NPC	1.10 (0.16-7.49)	0.922	<0.001	85.6%	2
HCC	1.91 (1.38-2.62)	<0.001	0.617	0	3
**Detection method**					
IHC	1.46 (1.01-2.11)	<0.05	<0.001	75.4%	10
Non-IHC	1.73 (1.40-2.13)	<0.001	0.601	0	5
**Follow-up period**					
<5	1.80 (1.29-2.51)	<0.05	0.509	0	4
≥5	1.58 (1.12-2.22)	<0.05	<0.001	81.7%	10
NA	0.77 (0.33-1.79)	0.545	NA	NA	1
**Method to estimate HR**					
Multivariate analysis	1.76 (1.31-2.37)	<0.001	<0.001	75.8%	13
Non-multivariate analysis	0.93 (0.23-3.68)	0.914	<0.001	92.3%	2
**Study quality**					
Low-risk	1.69 (1.39-2.05)	<0.001	0.705	0	7
Moderate-risk	1.45 (0.95-2.19)	0.084	<0.001	79.4%	8

DLBCL, diffuse large B-cell lymphoma; NPC, nasopharyngeal carcinoma; HCC, hepatocellular carcinoma; HR, hazard ratio; CI, confidence interval; IHC, only immunohistochemical; NA, not applicable.

### The relation between IDO1 expression and PFS and DFS in solid tumor patients

**Figure 3:**
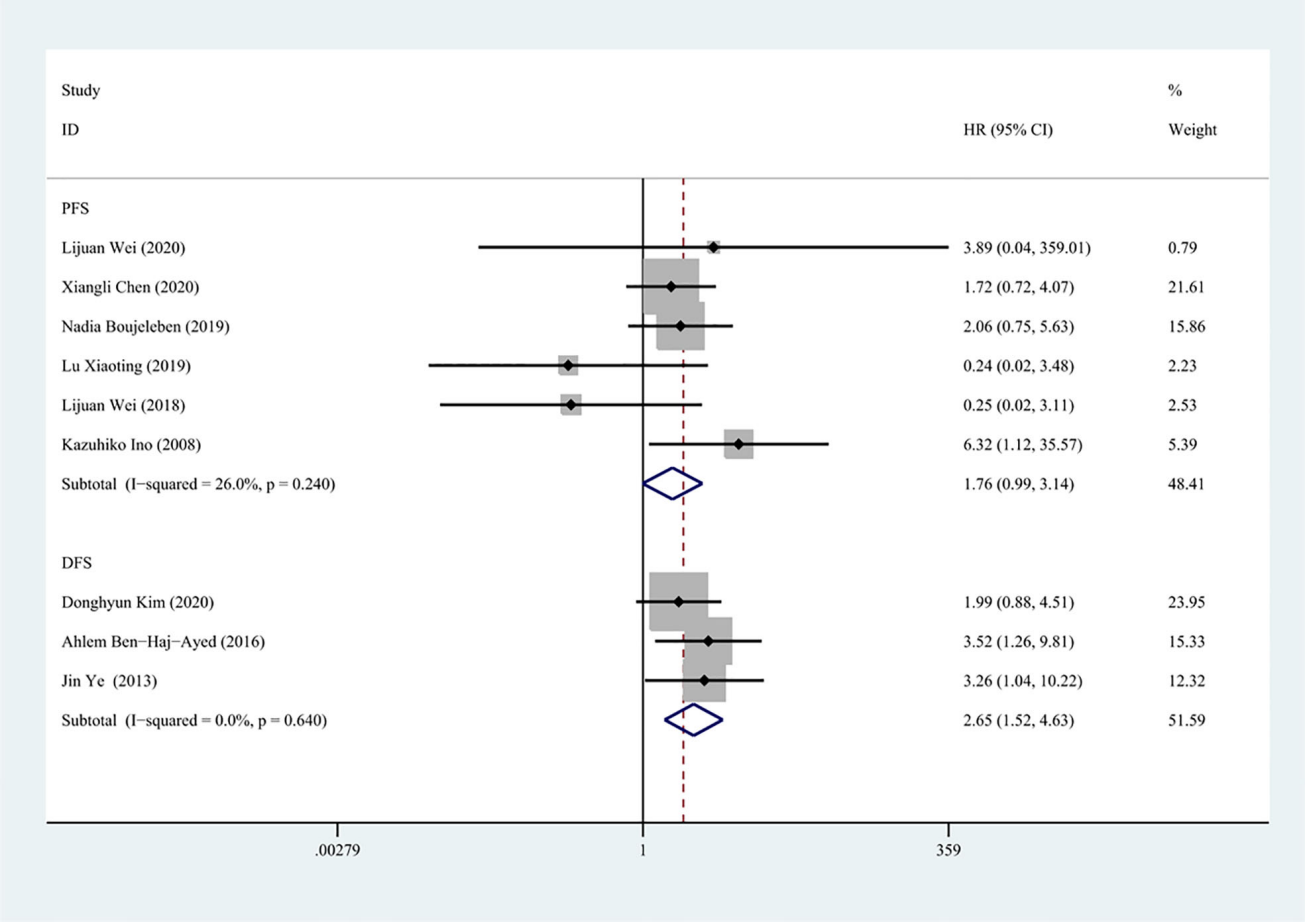
Forest plot of HR with 95% CI for correlation between expression of IDO1 and progression-free survival, disease-free survival.

There were totally six and three studies that reported the effects of IDO1 expression on PFS and DFS involved in the meta-analysis, respectively. A fixed-effect model was used to calculate the pooled HRs and 95% CIs, as the lower heterogeneity of PFS (I^2^ = 26.0%, P = 0.240) and DFS (I^2^ = 0, P = 0.640), respectively. Our results demonstrated that a high expression of IDO1 was associated with poor DFS (n = 327, HR = 2.65, 95% CI: 1.52–4.63, P = 0.001), while no significant correlation was found between IDO1 and PFS (n = 428, HR = 1.76, 95% CI: 0.99–3.14, P = 0.054) (**Figure 3**).

### Publication bias and sensitivity analysis

**Figure 4:**
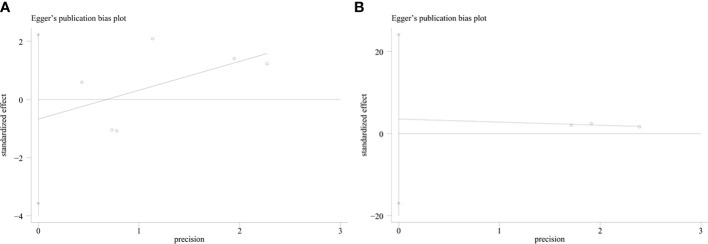
Result of Egger’s test. **(A)** Progression-free survival. **(B)** Disease-free survival.

**Figure 5:**
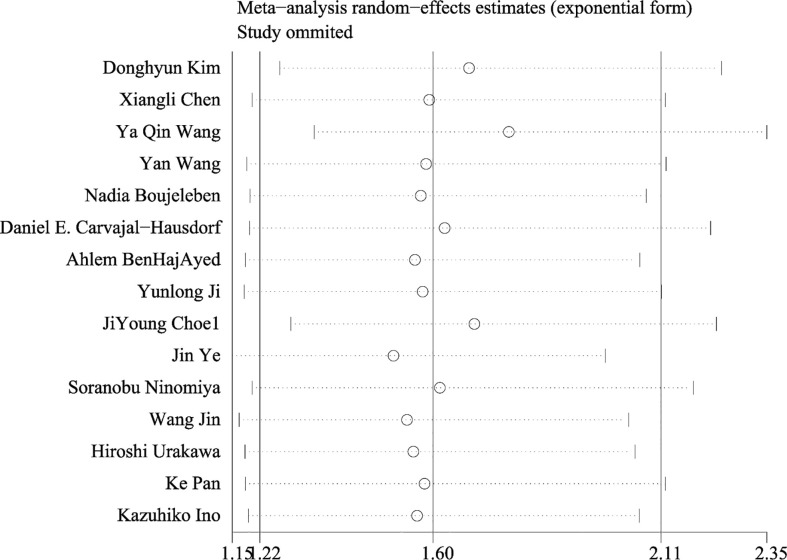
Consequence of sensitivity analysis of overall survival.

An evaluation of publication bias between studies was conducted using Egger’s test. Publication bias was found between IDO1 and OS (P = 0.002), but no publication bias was detected between IDO and PFS (P = 0.553), DFS (P = 0.273) (**Figure 4**). The trim-and-fill method was used to assess publication bias for OS. The overall effect was unchanged when four missing studies were added (HR = 1.418, 95% CI: 1.093–1.840, P = 0.009). Therefore, publication bias for OS had no impact on our results, which could be ignored. Sensitivity analysis showed that no single study had a significant impact on the conclusions of this meta-analysis (**Figure 5**), indicating that the results were stable.

## Discussion

IDO1 was first demonstrated by David Munn in 1998 to play crucial role in maintenance of maternal T-cell tolerance in the mouse placenta (54). Studies showed that IDO1 expression was strongly induced by interferon-γ in cell lines (55) and associated with lower CD3^+^ and CD8^+^ T lymphocyte infiltration and CD57^+^ NK-cell infiltration in tumor specimens (56), which could be the mechanism of immune escape. Many studies demonstrated that the expression of IDO1 in tumors was associated with prognosis (57, 58). However, the results were conflicting. Our meta-analysis included 2,168 patients with solid tumors from 18 studies. The results showed that a high expression of IDO1 was associated with a poor OS and DFS, which was consistent with previous meta-analyses (35, 36). Unexpectedly, no significant correlation was found between IDO1 expression and PFS. To the best of our knowledge, articles that have systematically evaluated the relationship between IDO1 expression and PFS in solid tumor are rare up to now, because of lack of enough data.

In this study, the heterogeneity of OS was obvious, which could be explained by several reasons. Firstly, 11 tumor types were included in this study and the role of IDO1 in different tumors may be inconsistent. Secondly, the cutoff value of IDO1 and treatment after surgery or biopsy could lead to the heterogeneity. To this end, subgroup analysis showed that age, gender, follow-up period, and study quality were possible reasons. The study of Stephane et al. showed that hyperprogressive disease was associated with a higher age and a worse OS in cancer patients treated by anti-PD-1/PD-L1, indicating a different immunological background in older patients (59). It was suggested that age could affect the immune microenvironment by altering the number, phenotype, and functions of neutrophils, macrophages, dendritic cells, and NK cells (60), further regulating IDO1 expression, which was similar to our study. Furthermore, Brandacher’s study showed that IDO-high expression was significantly correlated with better prognosis within the first 45 months, while the result was dramatically reversed after 45 months of follow-up (61), which attributed to the treatments during the follow-up period. This was why the follow-up period was a factor affecting heterogeneity in our study. In addition, the result of the subgroup analysis showed that the detection method could also lead to high heterogeneity, indicating the demand for more precise detection methods. Meanwhile, the studies involved in our meta-analysis were retrospective and the data identified were not comprehensive. It was supposed as the reason for the majority of studies suffering from a moderate risk of bias.

In a study of chemokine CXC motif ligand 12 (CXCL12), a prognosis factor similar to IDO1, a high CXCL12 expression was associated with a shorter OS in esophagogastric, pancreatic, or lung cancer, while the opposite was the case for breast cancer (62). Similarly, in our result, the relationship between IDO1 and PFS was different from OS and DFS, which may be explained by the included tumor types. Involving PFS, half of the included studies were about breast cancer. However, the exact cause of the different effects of IDO1 expression and outcome in breast was unclear. It was guessed that IDO1 could promote local invasion of cancer cells, which leads to a worse outcome in other cancers, but it was less affected in breast cancer, which was rarely fatal through local invasion unless metastasis occurs.

There were limitations in this meta-analysis. Firstly, we were unable to perform a subgroup analysis for each type of tumor, because of the limited number of included studies. Secondly, the cutoff values of IDO1 positivity and high expression were not completely consistent between studies, leading to the potential sources of heterogeneity. Thirdly, all the studies included were retrospective studies, lacking a prospective study. Additionally, the study was not registered in PROSPERO.

## Conclusion

The study showed that a high expression of IDO1 was associated with a poor OS and DFS in cancers. There was no significant correlation between IDO1 expression and PFS. IDO1 appears to be a promising therapeutic target as well as a prognostic predictor in different cancers. IDO1 inhibitors in monotherapy or in combination with other ICs will be the promising treatment. However, the immune regulator function of IDO1 in different types of cancer could not be completely consistent. Meanwhile, the exact mechanism of IDO1 in immune escape remains unclear. Future prospective studies are required to validate the relationship between expression of IDO1 and immune escape.

## Data availability statement

The original contributions presented in the study are included in the article/supplementary material. Further inquiries can be directed to the corresponding author.

## Author contributions

HZ searched and screened the literature according to the criteria, analyzed the data using Stata, and wrote the first draft of the manuscript. JL searched and screened the literature according to the criteria. QZ contributed to the conception and design of the study. All authors contributed to the manuscript revision and read and approved the submitted version.

## Conflict of interest

The authors declare that the research was conducted in the absence of any commercial or financial relationships that could be construed as a potential conflict of interest.

## Publisher’s note

All claims expressed in this article are solely those of the authors and do not necessarily represent those of their affiliated organizations, or those of the publisher, the editors and the reviewers. Any product that may be evaluated in this article, or claim that may be made by its manufacturer, is not guaranteed or endorsed by the publisher.
